# The Positive Impact of Vitamin D on Glucocorticoid-Dependent Skeletal Muscle Atrophy

**DOI:** 10.3390/nu13030936

**Published:** 2021-03-14

**Authors:** Mateusz Jakub Karnia, Daria Korewo, Dorota Myślińska, Ziemowit Maciej Ciepielewski, Monika Puchalska, Klaudia Konieczna-Wolska, Konrad Kowalski, Jan Jacek Kaczor

**Affiliations:** 1 Department of Physiology and Biochemistry, Gdansk University of Physical Education and Sport, Kazimierza Górskiego 1, 80-336 Gdansk, Poland; mateusz.karnia@awf.gda.pl (M.J.K.); daria.korewo@awf.gda.pl (D.K.); 2 Department of Animal and Human Physiology, Faculty of Biology, University of Gdansk, Wita Stwosza 59, 80-308 Gdansk, Poland; dorota.myslinska@biol.ug.edu.pl (D.M.); ziemowit.ciepielewski@biol.ug.edu.pl (Z.M.C.); nika.puchalska@gmail.com (M.P.); kkonieczna7@gmail.com (K.K.-W.); 3 Masdiag-Diagnostic Mass Spectrometry Laboratory, Stefana Żeromskiego 33, 01-882 Warsaw, Poland; konrad.kowalski@masdiag.pl

**Keywords:** dexamethasone, chronic stress, corticosterone, cold water immersion, soleus

## Abstract

(1) The study aimed to investigate whether vitamin D_3_ supplementation would positively affect rats with glucocorticoids-induced muscle atrophy as measured by skeletal muscle mass in two experimental conditions: chronic dexamethasone (DEX) administration and a model of the chronic stress response. (2) The study lasted 28 consecutive days and was performed on 45 male Wistar rats randomly divided into six groups. These included two groups treated by abdominal injection of DEX at a dose of 2 mg/kg/day supplemented with vegetable oil (DEX PL; *n* = 7) or with vitamin D_3_ 600 IU/kg/day (DEX SUP; *n* = 8), respectively, and a control group treated with an abdominal injection of saline (CON; *n* = 6). In addition, there were two groups of rats chronically stressed by cold water immersion (1 hour/day in a glass box with 1-cm-deep ice/water mixture; temperature ~4 °C), which were supplemented with vegetable oil as a placebo (STR PL; *n* = 9) or vitamin D_3_ at 600 IU/kg/day (STR SUP; *n* = 9). The last group was of sham-stressed rats (SHM; *n* = 6). Blood, soleus, extensor digitorum longus, gastrocnemius, tibialis anterior, and quadriceps femoris muscles were collected and weighed. The heart, liver, spleen, and thymus were removed and weighed immediately after sacrifice. The plasma corticosterone (CORT) and vitamin D_3_ metabolites were measured. (3) We found elevated CORT levels in both cold water-immersed groups; however, they did not alter body and muscle weight. Body weight and muscle loss occurred in groups with exogenously administered DEX, with the exception of the soleus muscle in rats supplemented with vitamin D_3_. Decreased serum 25(OH)D_3_ concentrations in DEX-treated rats were observed, and the cold water immersion did not affect vitamin D_3_ levels. (4) Our results indicate that DEX-induced muscle loss was abolished in rats supplemented with vitamin D_3_, especially in the soleus muscle.

## 1. Introduction

Skeletal muscle accounts for approximately 40% of body mass and is a major target organ for glucocorticoids (GCs). Under stressful or pathophysiological conditions such as starvation, cancer, or coldness, circulating GC levels are greatly increased. Likewise, the long-term or high-dose administration of synthetic GCs such as dexamethasone (DEX) may lead to decreased protein synthesis and increase proteolysis to generate amino acids to serve as precursors for hepatic gluconeogenesis. In the skeletal muscles, this leads to many adverse effects, particularly skeletal muscle atrophy and muscle weakness [1].

Moreover, GC activity may differ depending on whether it is administered externally or is of internal origin in relation to the stress response [2]. As data show, administering exogenous GCs like DEX to mimic a condition of physiological stress may not reflect a realistic condition to determine whether circulating GCs may attain the well-above peak levels observed during a stress response [3]. 

Vitamin D is considered to be a potent anti-catabolic compound [4,5]. Numerous studies suggest a positive role of vitamin D in sarcopenia prevention [6] or inhibition of muscle atrophy by suppression of forkhead box protein O1 (FOXO-1) transcriptional activity [7]. Some studies also imply that vitamin D has antioxidant potential both in the central nervous system [8] and skeletal muscles [9]. In work from our laboratory, we demonstrated that vitamin D deficiency induced protein peroxidation and atrophy in paraspinal muscle, and supplementation with vitamin D reversed those negative alterations [10,11]. On the other hand, the beneficial effect of vitamin D supplementation on skeletal muscle mass is questioned, and some research has indicated that vitamin D supplementation has little or no effect on muscle mass [12,13].

To our knowledge, there are no direct data on the effect of vitamin D_3_ on GC-induced muscle atrophy; however, there is some research focused on GC-induced osteoporosis [14] and the influence of the vitamin D analogs in the context of GC-dependent myopathy [15,16]. The exact effect that GCs have on vitamin D_3_ metabolism remains ambiguous. For instance, one study indicates that 1,25(OH)_2_D_3_ (calcitriol) stimulates (in human adipocytes) the expression of 11β-hydroxysteroid dehydrogenase type 1 (HSD11B1) [17]. The same research shows that calcitriol may act through a rapid, non-genomic mechanism that also stimulates GC release in adipocytes by increasing Ca^2+^ through 1,25-D3-membrane-associated rapid-response steroid binding (1,25-D3-MARRS) and, in consequence, increases the availability of GCs. Moreover, there is a report showing that calcitriol increases oxidative stress in cultured murine and human adipocytes [18]. Nevertheless, another study has shown a positive and highly selective type of activity of vitamin D, inducing oxidative stress only in malignant cells while sparing healthy cells [19]. 

In addition, serum vitamin D_3_ deficiency attenuated the protein content of vitamin D receptor (VDR), with a simultaneous elevated level of peroxidation markers of lipids and proteins in multifidus muscle [11]. There is also evidence linking vitamin D_3_ deficiency with GC administration. Data showed that patients who reported GC treatment were twice as likely to have vitamin D deficiency as compared to those without steroid use [20].

Furthermore, calcitriol is considered a true steroid hormone, and like GCs, it may exert several activities in many tissues and organs, demonstrating a synergistic effect in combined therapy [21]. An excellent example of this pharmacological approach is the use of calcitriol and DEX in patients with rheumatoid arthritis, where synovial fibroblast activation is abolished; this combination suppresses the expression of proinflammatory cytokines [22].

Vitamin D_3_ has been reported to suppress FOXO-1 transcriptional activity [7], and deficiency of vitamin D_3_ might induce skeletal muscle atrophy [10]. However, it is not clear whether vitamin D_3_ could prevent GC-induced muscle loss in vivo. Thus, the current study aimed to explore whether vitamin D supplementation attenuated detrimental changes as measured by the body and skeletal muscle weight in chronic DEX-administered rats. We also suspected that cold water immersion as a model of the chronic stress response (CSR) would induce an exogenous GC surge and, in consequence, cause similar deleterious effects. Therefore, supplementation with vitamin D would reverse the adverse effect induced by elevated GCs in the CSR.

## 2. Materials and Methods

### 2.1. Animals

The study was performed on 45 male Wistar rats (weighing approximately 300–400 g) obtained from the Medical University of Gdansk, Poland. For the whole experiment the animals were housed 3–4 per cage with food and water provided ad libitum, with a 12-h light/dark cycle and controlled environmental conditions: temperature 22 °C and humidity 55%. Studies were conducted with the consent of the Local Bioethics Committee in Bydgoszcz, Poland (No. 12/2019), according to European guidelines. 

### 2.2. Study Design

Two weeks before the experiment, animals were handled to acclimate and minimalize stress. Next, rats were randomly divided into 6 groups. Two of these groups were treated with an abdominal injection of dexamethasone (Dexamethasone D4902, Sigma–Aldrich, MN, USA) at 2 mg/kg/day supplemented with vegetable oil (DEX PL; *n* = 7), or vitamin D_3_ (DEX SUP; *n* = 8). The control group was treated using an abdominal injection of saline (CON; *n* = 6). Two groups of rats chronically stressed by cold water immersion were given supplementation with vegetable oil as a placebo (STR PL; *n* = 9) or vitamin D_3_ (STR SUP; *n* = 9). The last group comprised sham-stressed (warm water-immersed) rats (SHM; *n* = 6). 

The STR PL and STR SUP groups were exposed over 28 days to chronic stress by isolation in the glass box (21 × 15 × 30 cm) for 1 hour per day with a 1-cm-deep ice/water mixture (0–4 °C), and the SHM group was placed in sham stress conditions (warm water (35 °C)). The animals from the STR PL and DEX PL groups received oral administration of the vegetable oil as a placebo, and the STR SUP and DEX SUP groups were supplemented with vitamin D_3_ at 600 IU/kg (Juvit D3, PPF HASCO-LEK. SA., Poland) for 28 consecutive days. 

### 2.3. Blood Collection

Blood was collected at 2-time points, prior to and after 28 days of the experiment. Blood was taken from the tail vein during isoflurane anesthesia. Samples were centrifuged at 2000× *g* for 10 min at 4 °C. Serum samples were separated and stored at −80 °C until later analysis. 

### 2.4. Tissue Collection

Soleus, extensor digitorum longus, gastrocnemius, tibialis anterior, and quadriceps femoris muscles were collected from both hind limbs, weighed, and snap-frozen in liquid nitrogen and kept at −80 °C for later analysis. The heart, liver, spleen, and thymus were excised and weighed immediately after sacrifice.

### 2.5. Biochemical Analysis

#### 2.5.1. Corticosterone Level

According to the manufacturer’s instructions, the plasma corticosterone level was determined with a Corticosterone rat/mouse ELISA Kit (DEV9922, Demeditec Diagnostics GmBH, Kiel, Germany). The concentration of CORT was expressed in nanograms per milliliter of plasma.

#### 2.5.2. Vitamin D-25(OH)D_3_, 3-epi-25(OH)D_3_, 25(OH)D_2_, 24.25(OH)_2_D_3_ Levels

Analysis of the vitamin D_3_ metabolite levels was performed using the isotope dilution method by the liquid chromatography coupled with tandem mass spectrometry technique (LC-MS/MS). Samples were prepared and analyzed using the Eksigent ExionLC analytical HPLC system with a CTC PAL autosampler (Zwinger, Switzerland) coupled with QTRAP^®^ 4500 MS/MS system (Sciex, Framingham, MA, USA) according to the procedure described previously [23].

### 2.6. Statistical Analysis

All statistical analyses were performed using the GraphPad Prism 8.3 software program (GraphPad Software, CA, USA). The results are expressed as mean ± SD. The differences between groups were tested using one-way ANOVA followed by the Tukey post-hoc test; *p*-values less than 0.05 were considered statistically significant.

## 3. Results

### 3.1. Plasma Corticosterone (CORT) Level

**Figure 1:**
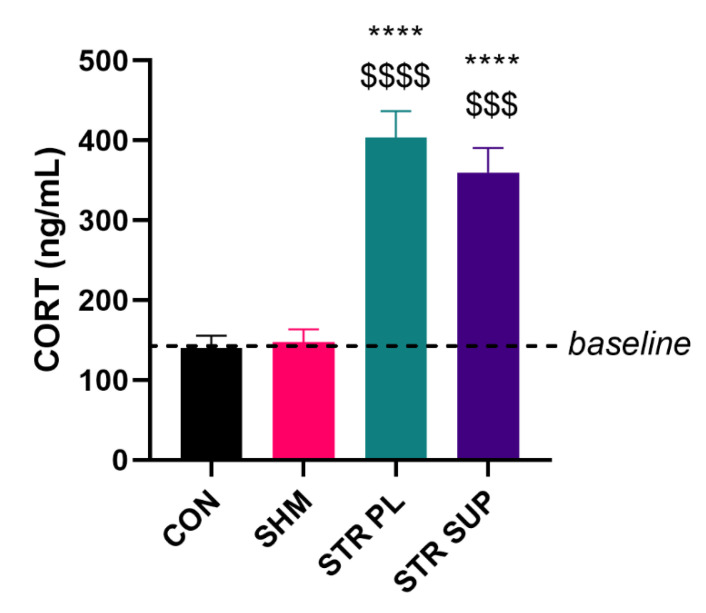
The level of corticosterone (CORT) in plasma. Results are expressed as mean ± SEM. CON (*n* = 6), SHM (*n* = 6), STR PL (=9), STR SUP (*n* = 9), **** *p* < 0.0001 vs. CON, $$$$ *p* < 0.0001 vs. SHM, $$$ *p* < 0.001 vs. SHM. CON: control group; SHM: sham cold water immersion group; STR PL: cold water immersion group supplemented with placebo; STR SUP: cold water immersion group supplemented with vitamin D_3_.

As was expected, cold water immersion treatment caused a significant induction in hypothalamic–pituitary–adrenal (HPA) axis activation and a CORT surge into the blood flow. Plasma CORT level significantly increased in both (placebo and supplemented) stressed groups of rats. The levels were 403.54 ± 49.73 in the stressed placebo (STR PL) and 359.67 ± 46.32 ng/mL in the stressed supplemented with vitamin D_3_ (STR SUP) groups, respectively. There were no differences from the baseline in the control sham-stressed (SHM) rats. In order to assess the correctness of the selection of sham stress conditions, we also determined the CORT level in the control (CON) group, and no changes in that group were observed (Figure 1).

### 3.2. Vitamin D Biochemical Analysis

#### 3.2.1. Plasma Vitamin D_3_ Metabolite Levels in DEX-Treated Rats

**Figure 2:**
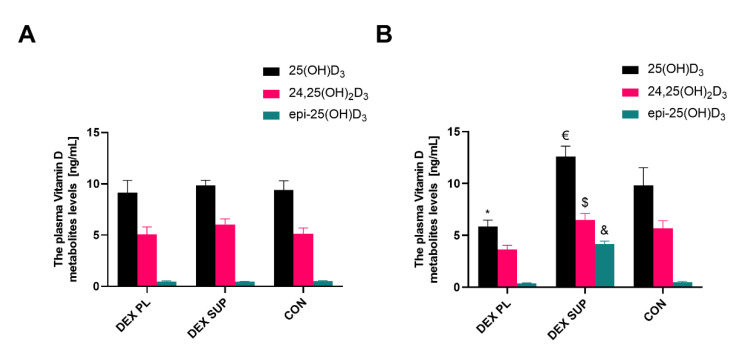
The plasma vitamin D metabolite levels in DEX-treated rats at baseline (**A**) and the end of the experiment (**B**). Results are expressed as mean ± SEM. DEX PL (*n* = 7), DEX SUP (*n* = 8), CON (*n* = 6), * *p* < 0.05 vs. CON, $ *p* < 0.01 vs. DEX PL, € *p* < 0.001 vs. DEX PL, & *p* < 0.0001 vs. DEX PL; vs. CON. DEX PL: dexamethasone-treated group supplemented with placebo; DEX SUP: dexamethasone-treated group supplemented with vitamin D_3_; CON: control group.

After four weeks of the experiment, the level of 25(OH)D_3_ significantly differed between the groups. As we expected, the highest concentration of 25(OH)D_3_ was observed in the supplemented group. However, the dexamethasone-treated supplemented with vitamin D_3_ (DEX SUP) group differed only from the dexamethasone-treated placebo (DEX PL) group, not from the CON group (the values were 12.59 ± 2.87 in the DEX SUP, 5.87 ± 1.62 in the DEX PL, and 9.85 ± 4.12 ng/mL in the CON groups, respectively) (Figure 2B). 

In the CON group within the experiment, the results were relatively homogeneous and transparent. No changes were observed in either 25(OH)D_3_ or 24,25(OH)_2_D_3_ during the experiment, proving the correct blinding in the group and the lack of vitamins D_3_ or D_2_ in the feed.

In the DEX PL group we observed a significant reduction in the bioavailable form of vitamin D_3_-25(OH)D_3_. Besides, no catabolic mechanisms were activated: the values for epi-25(OH)D_3_ and 24,25(OH)_2_D_3_ were lower, but their ratio to 25(OH)D_3_ remained practically unchanged between time points. This could reflect a clinical case of high demand for vitamin D_3_ and its heavy consumption to defend muscles against atrophy. Circulating 25(OH)D_3_ is absorbed first. The rapid mobilization of vitamin D_3_ from the body’s fat reserves is not visible here. The appearance of a large pool of endogenous vitamin D_3_ would be manifested in changes in the ratio of 25(OH)D_3_ to epi-25(OH)D_3_ and 24,25(OH)_2_D_3_, respectively (as in the DEX SUP group, where vitamin D_3_ was obtained exogenously) (Table 1).

Additionally, the lack of an increase in 24,25(OH)_2_D_3_ and significant changes in the 25(OH)D_3_:24,25(OH)_2_D_3_ ratio, which works on the principle of feedback with 1,25(OH)_2_D_3_, supports the thesis that a rapid “on-going” consumption of bioavailable vitamin D_3_ to protect against muscle atrophy in that particular group occurred (Table 1).

#### 3.2.2. Plasma Vitamin D_3_ Metabolite Levels in Stressed Rats

**Figure 3:**
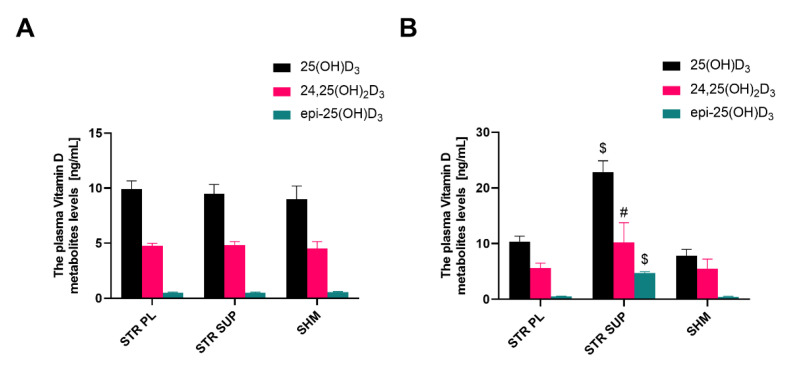
The plasma vitamin D metabolite levels in stressed rats at baseline (**A**) and the end of the experiment (**B**). Results are expressed as mean ± SEM. STR PL (*n* = 9), STR SUP (*n* = 9), SHM (*n* = 6), # *p* < 0.01 vs. STR PL; vs. SHM, $ *p* < 0.0001 vs. STR PL; vs. SHM. STR PL: cold water immersion group supplemented with placebo; STR SUP: cold water immersion group supplemented with vitamin D_3_; SHM: sham cold water immersion group.

Similar to the DEX-treated rats, a significantly higher concentration of 25(OH)D_3_ was observed only in supplemented group (22.89 ± 6.02) as compared with the STR PL (10.36 ± 2.92) and the SHM groups (7.84 ± 2.80 ng/mL). In addition, there was no effect of warm water immersion on native vitamin D_3_ concentration and metabolism. Additionally, in the STR PL group, the results were the same as for the SHM groups. This also means that cold water immersion had no effect on native vitamin D_3_ concentration and metabolism (Figure 3B).

Considering the above, the STR SUP group may be treated as a positive control of vitamin D_3_ supplementation. A fully statistically significant increase in both 25(OH)D_3_, epi-25(OH)D_3_, and 24,25(OH)_2_D_3_ shows that the body responds correctly to the supplementation. A particularly clear difference is visible for epi-25(OH)D_3_, and the 25(OH)D_3_:epi-25(OH)D_3_ ratio. It is worth noting that in rodents (unlike humans), epimerization is the main “default” path of catabolism in response to exogenous vitamin D_3_ (Table 2). 

### 3.3. Morphological Analysis

#### 3.3.1. Body and Skeletal Muscle Mass in DEX-Treated Rats

**Figure 4:**
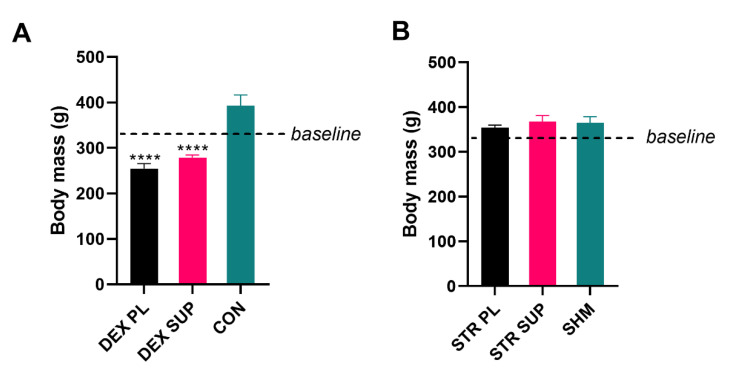
The body mass at the end of the experiment in DEX-treated (**A**), and stressed (**B**) rats. Results are expressed as mean ± SEM. DEX PL (*n* = 7), DEX SUP (*n* = 8), CON (*n* = 6), STR PL (*n* = 9), STR SUP (*n* = 9), SHM (*n* = 6), **** *p* < 0.0001 vs. CON. DEX PL: dexamethasone-treated group supplemented with placebo; DEX SUP: dexamethasone-treated group supplemented with vitamin D_3_; CON: control group; STR PL: cold water immersion group supplemented with placebo; STR SUP: cold water immersion group supplemented with vitamin D_3_; SHM: sham cold water immersion group.

The DEX-treated groups presented weight loss throughout the experiment as compared with the CON group (Figure 4A). 

**Figure 5:**
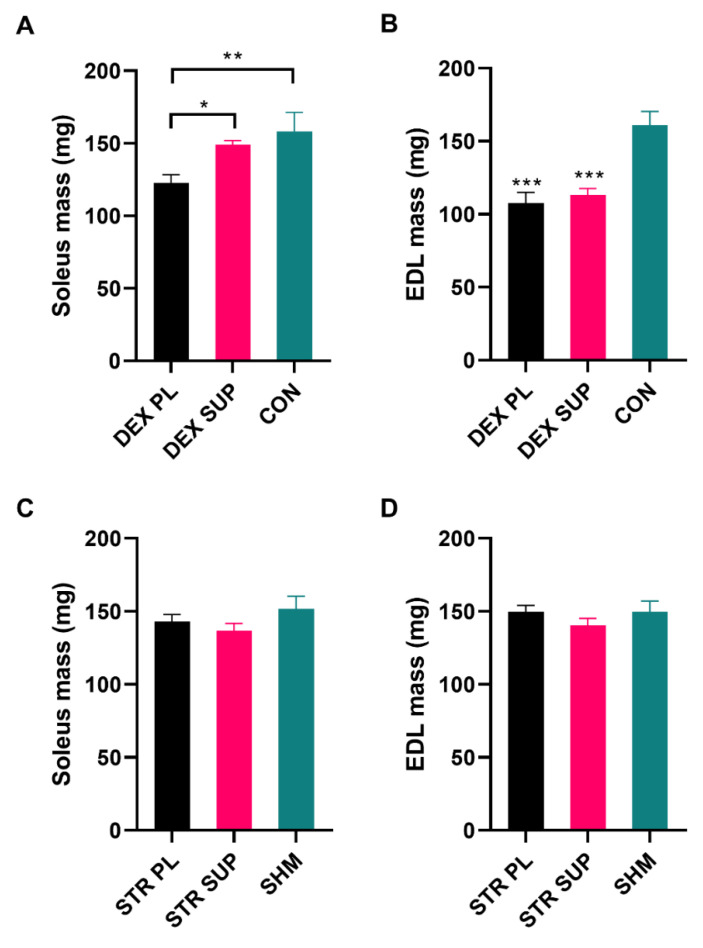
The SOL and EDL muscles mass at the end of the experiment in DEX-treated (**A**,**B**), and stressed (**C**,**D**) rats. Results are expressed as mean ± SEM. DEX PL (*n* = 7), DEX SUP (*n* = 8), CON (*n* = 6), STR PL (*n* = 9), STR SUP (*n* = 9), SHM (*n* = 6), * *p* < 0.05 vs. DEX SUP, ** *p* < 0.01, *** *p* < 0.001 vs. CON. DEX PL: dexamethasone-treated group supplemented with placebo; DEX SUP: dexamethasone-treated group supplemented with vitamin D_3_; CON: control group; STR PL: cold water immersion group supplemented with placebo; STR SUP: cold water immersion group supplemented with vitamin D_3_; SHM: sham cold water immersion group.

To investigate skeletal muscle loss, the weights of the soleus (SOL), extensor digitorum longus (EDL) (Figure 5A,B), tibialis anterior, gastrocnemius, and quadriceps femoris muscles were measured immediately after excision (Table 3). Although supplementation with vitamin D_3_ did not prevent this DEX-induced body mass loss, we found statistically significant differences in SOL muscle mass between the DEX PL and the DEX SUP groups (122 ± 15, and 149 ± 9 mg, respectively; *p* < 0.05). Additionally, there was no difference between the DEX SUP and the CON group (Figure 5A). 

Regarding the EDL, we observed a significant reduction in muscle mass in both DEX-treated groups compared with the CON group (107 ± 19 DEX PL, 113 ± 13 DEX SUP, and 161 ± 21 mg CON, respectively; *p* < 0.001). Nevertheless, there were no differences between the supplemented and placebo groups. These results suggest that vitamin D_3_ supplementation influences DEX-induced muscle loss, but only in red, not white muscle, and may preserve red muscle against the chronic DEX-induced muscle loss. The masses of the other muscles were consistent and homogeneous. There was a clear and highly statistically significant reduction in all muscle mass in both DEX-treated groups regardless of supplementation with vitamin D_3_ or placebo.

**Figure 6:**
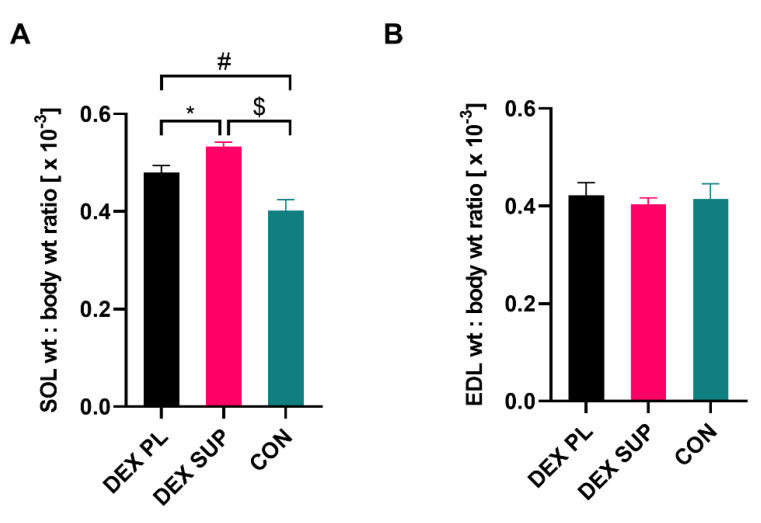
Mean muscle weight: body weights ratio in DEX-treated rats in SOL (**A**), and EDL (**B**). Results are expressed as mean ± SEM. DEX PL (*n* = 7), DEX SUP (*n* = 8), CON (*n* = 6), * *p* < 0.05, # *p* < 0.01, $ *p* < 0.0001. DEX PL: dexamethasone-treated group supplemented with placebo; DEX SUP: dexamethasone-treated group supplemented with vitamin D_3_; CON: control group; EDL: extensor digitorum longus; SOL: soleus.

No significant difference was found in the muscle:body weight ratios of CON and DEX-treated rats in EDL (Figure 6B). However, the SOL muscle weight to body weight ratio significantly increased in DEX-treated rats compared to the CON group (Figure 6A). This showed not only that there was a relative sparing of SOL muscles as compared with EDL within the experiment, but also sparing of muscle tissue in general relative to other body components. In particular, SOL sparing affected the DEX SUP group, suggesting a protective role of vitamin D_3_ supplementation.

#### 3.3.2. Body and Skeletal Muscle Mass in Stressed Rats

Interestingly, despite highly statistically significant CORT release (Figure 1), we did not observe body weight changes in the group of rats subjected to cold water immersion (Figure 4B). Furthermore, no statistically significant differences were observed between the groups in SOL (143 ± 15, 136 ± 16, and 152 ± 21 mg in the STR PL, STR SUP, and SHM groups, respectively) and EDL (STR PL 149 ± 13, STR SUP 140 ± 15, SHM 149 ± 18 mg) muscle mass (Figure 5C,D).

Although we did not observe any statistically significant differences between groups in stressed rats with regard to muscle mass (Table 4), as opposed to the DEX SUP group, stressed rats supplemented with vitamin D_3_ had the lowest (statistically insignificant) ratios in both muscles among groups (Table 5). Considering the lack of body reduction in stressed groups, that result supports the thesis that the used stress model promotes adipose tissue gain (in turn, an enlarged adipose mass may serve as a reservoir for vitamin D).

#### 3.3.3. Internal Organ Mass in DEX-Treated Rats

Heart and liver weights did not differ significantly between groups. Nevertheless, differences in the weight of the organs of the lymphatic system were observed. Thus, spleen weight was the lowest of the two DEX-treated groups. Besides, there was a statistically significant reduction in thymus weight in both DEX-treated groups compared to the control group (0.090 ± 0.053, 0.148 ± 0.091 vs. 0.372 ± 0.054 g in the DEX PL, DEX SUP, and CON group, respectively) (Table 6).

#### 3.3.4. Internal Organs Mass in Stressed Rats

Similarly to DEX-treated groups, in stressed rats we did not observe any statistically significant changes in heart and liver mass. Nevertheless, we observed significantly lower thymus weight in the stressed animals treated with placebo, but not in the vitamin D_3_-supplemented group compared with the SHM group (0.233 ± 0.045 in the STR PL, 0.305 ± 0.101 in the STR SUP, and 0.348 ± 0.082 g in the SHM groups, respectively; *p* < 0.05), which suggest that vitamin D_3_ supplementation may protect thymus against degeneration caused by GCs, particularly in the chronic stress condition (Table 7).

## 4. Discussion

The role of vitamin D_3_ within the skeletal muscle is in the scope of interest of many researchers. Although the beneficial effect of vitamin D_3_ on skeletal muscle mass remains unclear, some research suggests that vitamin D_3_ may prevent skeletal muscle loss and atrophy [24], while other papers indicate that vitamin D_3_ supplementation has little or no effect on muscle mass [12,13]. We found that chronic DEX treatment decreased serum 25(OH)D_3_ concentrations. We also showed that GC-induced body and muscle loss are presented only in exogenously administered DEX. Our results indicate that DEX-induced muscle loss is abolished in rats supplemented with vitamin D_3_ but only in SOL muscle. Based on previously published data [25], we assumed that a similar effect should be observed in the CSR conditions. However, instead of HPA axis activation and CORT surge, we did not note any changes in the body and muscle weight. Moreover, the cold water immersion had no effect on the native vitamin D_3_ levels despite the highly and statistically significant elevated level of circulating CORT in stressed rats.

### 4.1. Chronic Stress Response and Its Effect on the Body, Skeletal Muscle, and Organ Mass

The chronic stress response model used in the experiment was based on the procedure that combines physical (low temperature) and psychological stress (impossibility to escape and isolation). The obtained data show that the CSR was successfully induced, and the level of circulating CORT level highly increased from the baseline compared to the SHM and CON groups. The CORT concentration at 400 ng/mL levels corresponded with the results obtained in our other CSR experiment [26] and with the works based on the cold water immersion model [27,28]. 

Despite the physiologically significant CORT surge, we did not observe any body and muscle weight changes in the supplemented and placebo groups. Interestingly, in an experiment conducted by Nishida and coworkers, no changes in SOL and EDL muscle mass were observed even during DEX treatment (5 days, dose 600 µg/kg) [29]. Our recent work [25,26] showed that the CSR and increased CORT level do not have to accompany changes in body and muscle weight (data not shown); nevertheless, an increased level of atrogin-1 was observed. Furthermore, we assume that to explain this phenomenon in these particular conditions, it is necessary to consider intramuscular fat stores in rats from groups exposed to the chronic cold water immersion [30,31]. In support of this thesis, we can mention that, during autopsy, an increased amount of total fat mass and adipose tissue browning in two reference points (the suprascapular and supraspinal areas) was observed [32] (data not shown). 

Lastly, the classically indicated “stress triad” (a term proposed by Selye) assumes that in first reaction to stress there are three visible changes: adrenal enlargement, atrophy of the thymus, and hemorrhagic gastric erosions [33]. A partly similar observation was made in our study, where thymus weight significantly decreased in rats from the STR PL, while no changes in the STR SUP group were observed (Table 7). 

### 4.2. Dexamethasone Treatment and Its Effect on the Body, Skeletal Muscle, and Organ Mass

Our results show that chronic DEX administration reduced body weight in both DEX-treated groups (23% and 17% in DEX PL and DEX SUP, respectively), and supplementation with vitamin D_3_ did not attenuate this effect in a statistically significant manner. Moreover, in line with Selye’s assumptions, thymus and spleen degradation was also observed in both DEX-treated groups. Despite the lack of bodyweight preservation in the DEX SUP group, SOL muscle-sparing in the vitamin D_3_ supplemented group was observed. Muscle atrophy is a major adverse effect observed after DEX administration; however, the exact mechanism responsible for DEX-induced muscle atrophy is not well understood. Some data show that DEX acts mainly on muscles containing type II fast-twitch fibers as compared with type I slow-twitch fibers [34,35]. Similar observations were made by Krug and coworkers, where DEX treatment reduced flexor hallucis longus and tibialis anterior mass without SOL mass loss [36]. Our results are only partially in line with previous observations. Chronic DEX administration caused massive muscle loss in both red and white muscles, sparing only SOL in the group supplemented with vitamin D_3_. 

### 4.3. The Potential Protective Role of Vitamin D_3_ in Skeletal Muscle in GC-Induced Muscle Loss Conditions

Our results show the massive consumption of vitamin D_3_ in DEX-treated rats to defend muscles against atrophy, especially in the DEX PL group. Circulating 25(OH)D_3_ was utilized first without the rapid mobilization of vitamin D_3_ from the body’s fat reserves. Furthermore, in the DEX SUP group, the rapid “on-going” consumption of circulating vitamin D_3_ to protect against muscle atrophy was visible. 

Several clinical works highlight the positive aspects of vitamin D supplementation in many diseases. According to the Endocrine Society Clinical Practice Guideline on the Prevention of Vitamin D Deficiency, concentrations of 25(OH)D_3_ from 21 to 29 ng/mL (52.5–72.5 nmol/L) in serum are insufficient, and levels lower than 20 ng/mL (50 nmol/L) are considered to reflect deficiency [37]. As the data show, normalizing the level of circulating vitamin D enhances the reduction of systemic inflammation markers and intensity of pain in low back patients [37]. Another study shows that supplementation for six months reduced oxidative protein damage, decreased pain, improved quality of life, and improved grip strength and physical performance in osteoarthritis patients [38]. Vitamin D supplementation is also increasingly used in the prevention and therapy of sarcopenia [5,6] and neuromuscular diseases [39,40]. Moreover, studies show that vitamin D deficiency results in a more severe course of SARS-COV2 virus infection, and vitamin D supplementation is one of the proposed strategies for relieving symptoms of the disease [41]. Additionally, novel findings suggest that the early use of DEX could reduce duration of mechanical ventilation and overall mortality in patients with established moderate-to-severe acute respiratory distress syndrome (ARDS) in SARS-COV2 infected patients [42]. The low potential risk of vitamin D overdose (using the standard proposed dose appropriate to age, e.g., 2000 IU for an adult) [43,44], and the cost-effective aspect of vitamin D supplementation [45] should be considered in support of its use for the treatment of various diseases. According to knowledge about several common regulatory pathways which vitamin D and DEX share [46], using these therapeutics in combination may prove to be the most effective known strategy against SARS-COV and also other diseases (i.e., rheumatoid arthritis [22]) due to improved function of the immune system and minimized side effects of DEX-treatment.

However, the mechanism of vitamin D-mediated changes in skeletal muscle is not fully elucidated. It is known that vitamin D_3_ acts mainly via specific binding to an intracellular VDR, interacting with specific nucleotide sequences of over 60 target genes [47]. Numerous data show that the interaction between GCs and VDR occurs. Therefore, in the study conducted by Hidalgo and coworkers, induction with GCs increased VDR transcription in squamous cell carcinoma VII (SCC) to the level of 4–6 fold higher compared to the control group [48].

Additionally, a novel mitochondrial localization of VDR has been described. Data show that VDR influences mitochondrial respiration reduction and serves in reprogramming in cell metabolism toward the biosynthetic pathways [49,50]. This underlines the importance of mitochondria as the hub linking the processes such as cell development and atrophy inhibition in skeletal muscles. Other studies suggest that VDR plays a fundamental regulatory role in skeletal muscle mitochondrial function [51]. Moreover, the cooperative action of vitamin D_3_ and GCs in modulating gene expression was presented [17] which implies the potential reduction of the adverse effects of GC excess (during vitamin D_3_ supplementation) [52]. In our study, this phenomenon is partly confirmed because SOL muscle consists of predominantly slow-oxidative fibers, with a larger pool of mitochondria, and EDL is mainly formed of fast-glycolytic muscle fibers [53]. Our results show that in DEX-induced atrophy rats, SOL muscle is sparing in both absolute (Figure 5A) and relative values (Figure 6A). In summary, the main explanation for such a massive decrease in vitamin D concentration with partial protection against atrophy is the supposition that skeletal muscle cells overexpress VDR under both atrophy and hypertrophy conditions [54].

## 5. Conclusions

Our findings show that despite the elevated circulating CORT in cold water-immersed rats, no body and muscle weight changes were observed in either the vitamin D_3_-supplemented or placebo groups. We found that chronic DEX treatment decreased serum 25(OH)D_3_ concentrations, and cold water immersion had no effect on native vitamin D_3_ levels. Moreover, body weight and muscle loss occurred concomitantly only with exogenously administered DEX. Our results indicate that DEX-induced muscle loss was abolished in rats supplemented with vitamin D_3_, but only concerning the SOL muscle. The massive consumption of endogenous vitamin D_3_ was caused by an attempt to protect against muscle loss in DEX-treated rats. The additional supply of exogenous vitamin D_3_ in the DEX SUP group supports that this rapid “on-going” utilization of circulating vitamin D_3_ was accompanied by the protection of muscle atrophy. Our findings show that DEX treatment should be combined with vitamin D_3_ supplementation since the long-term treatment of DEX leads to a sharp reduction in vitamin D_3_ levels. Moreover, as a consequence, this may contribute to the adverse effects of DEX treatment alone. 

### Study Limitation

The findings of the present study are limited because the experiments were focused on visible morphological changes and not on the mechanism(s) responsible for the effects of vitamin D_3_ supplementation on GC-induced muscle atrophy per se. Nevertheless, we found that supplementation with vitamin D_3_ reduced the adverse effects on muscle loss in chronic DEX-treated rats, which indicates that further studies are needed to clarify the possible molecular mechanism(s) explaining this phenomenon.

## Figures and Tables

**Table 1:** The 25(OH)D_3_ and its metabolite ratios at baseline and the end of the experiment in DEX-treated rats.

Group	25(OH)D_3_: 24,25(OH)_2_D_3_ Ratio		25(OH)D_3_: epi-25(OH)D_3_ Ratio	
	Baseline	after	Baseline	after
DEX PL (*n* = 7)	1.83 ± 0.18	1.65 ± 0.30	20.27 ± 3.08	16.04 ± 2.51 *
DEX SUP (*n* = 8)	1.70 ± 0.40	2.17 ± 1.00	21.97 ± 3.22	3.11 ± 0.88 #
CON (*n* = 6)	1.88 ± 0.48	1.68 ± 0.30	20.33 ± 6.17	20.62 ± 4.63

Results are expressed as mean ± SD. * *p* < 0.05 vs. CON, # *p* < 0.0001 vs. DEX PL; vs. CON.

**Table 2:** The 25(OH)D_3_ and its metabolite ratios at baseline and the end of the experiment in stressed rats.

Group	25(OH)D_3_: 24,25(OH)_2_D_3_ Ratio		25(OH)D_3_: epi-25(OH)D_3_ Ratio	
	Baseline	after	Baseline	after
STR PL (*n* = 9)	2.10 ± 0.40	1.82 ± 0.36	20.20 ± 3.21	20.63 ± 1.75
STR SUP (*n* = 9)	1.94 ± 0.23	2.43 ± 0.92 &	19.34 ± 2.71	4.96 ± 1.34 $
SHM (*n* = 6)	1.99 ± 0.22	1.40 ± 0.21	16.88 ± 3.79	17.25 ± 4.99

Results are expressed as mean ± SD, & *p* < 0.01 vs. SHM, $ *p* < 0.0001 vs. STR PL; vs. SHM.

**Table 3:** Body and skeletal muscles mass at the end of the experiment in DEX-treated rats.

Group	Basal Body Mass (g)	Final Body Mass (g)	Tibialis Anterior (g)	Gastrocnemius (g)	Quadriceps Femoris (g)
DEX PL (*n* = 7)	332.57 ± 23.62	254.86 ± 28.06 $	0.39 ± 0.11 $	1.28 ± 0.20 $	1.41 ± 0.24 $
DEX SUP (*n* = 8)	337.38 ± 24.54	279.13 ± 14.55 $	0.46 ± 0.04 $	1.42 ± 0.16 $	1.58 ± 0.26 $
CON (*n* = 6)	335.60 ± 48.20	393.00 ± 52.05	0.75 ± 0.05	2.24 ± 0.18	2.44 ± 0.29

Results are expressed as mean ± SD. $ *p* < 0.0001 vs. CON.

**Table 4:** Body and skeletal muscle mass at the end of the experiment in stressed rats.

Group	Basal Body Mass (g)	Final Body Mass (g)	Tibialis Anterior (g)	Gastrocnemius (g)	Quadriceps Femoris (g)
STR PL (*n* = 9)	324.44 ± 22.10	353.89 ± 17.32	0.69 ± 0.05	2.10 ± 0.16	2.71 ± 0.29
STR SUP (*n* = 9)	337.78 ± 41.77	367.67 ± 40.43	0.67 ± 0.07	2.15 ± 0.25	2.70 ± 0.45
SHM (*n* = 6)	329.00 ± 33.66	364.83 ± 33.52	0.71 ± 0.07	2.16 ± 0.33	2.79 ± 0.48

Results are expressed as mean ± SD.

**Table 5:** Mean muscle weight: body weights ratio in stressed rats.

Group	SOL Weight: Body Weight Ratio (×10^−3^)	EDL Weight: Body Weight Ratio (×10^−3^)
STR PL (*n* = 9)	0.403 ± 0.036	0.423 ± 0.039
STR SUP (*n* = 9)	0.374 ± 0.048	0.383 ± 0.038
SHM (*n* = 6)	0.415 ± 0.035	0.410 ± 0.050

Results are expressed as mean ± SD.

**Table 6:** Internal organ mass at the end of the experiment in DEX-treated rats.

Group	Heart (g)	Liver (g)	Spleen (g)	Thymus (g)
DEX PL (*n* = 7)	0.83 ± 0.11	10.26 ± 0.93	0.25 ± 0.04 $	0.090 ± 0.053 $
DEX SUP (*n* = 8)	0.92 ± 0.09	11.08 ± 1.34	0.30 ± 0.03 $	0.148 ± 0.091 #
CON (*n* = 6)	0.92 ± 0.10	11.88 ± 2.16	0.65 ± 0.06	0.372 ± 0.054

Results are expressed as mean ± SD, # *p* < 0.001 vs. CON, $ *p* < 0.0001 vs. CON.

**Table 7:** Internal organs mass at the end of the experiment in stressed rats.

Group	Heart (g)	Liver (g)	Spleen (g)	Thymus (g)
STR PL (*n* = 9)	0.94 ± 0.05	11.22 ± 0.77	0.63 ± 0.08	0.233 ± 0.045 *
STR SUP (*n* = 9)	0.95 ± 0.06	10.77 ± 1.31	0.66 ± 0.07	0.305 ± 0.101
SHM (*n* = 6)	0.89 ± 0.09	11.48 ± 1.46	0.60 ± 0.11	0.348 ± 0.082

Results are expressed as mean ± SD. * *p* < 0.05 vs. SHM.

## Data Availability

The datasets used and/or analyzed during the current study are available from the corresponding author on reasonable request.

## Abbreviations

CORT	Corticosterone
CSR	Chronic stress response
DEX	Dexamethasone
FOXO-1	Forkhead box protein O1
GCs	Glucocorticoids
HPA	Hypothalamic–pituitary–adrenal axis
HSD11B1	11β–hydroxysteroid dehydrogenase type 1
VDR	Vitamin D receptor
1,25–D3-MARRS	1,25D3–membrane associated, rapid response steroid–binding
